# Sensitivity and Specificity of Hepatitis B Virus Screening via Rapid Immunoassay Chromatographic Test

**DOI:** 10.7759/cureus.12909

**Published:** 2021-01-25

**Authors:** Rukhsana Saboor Soomro, Iftikhar Ali Shah, Abdul Saboor, Aman Ullah B Bhutto, Sidra Memon

**Affiliations:** 1 Pathology, Ghulam Muhammad Mahar Medical College Hospital, Sukkur, PAK; 2 Internal Medicine, Ghulam Muhammad Mahar Medical College Hospital, Sukkur, PAK; 3 Urology, Ghulam Muhammad Mahar Medical College Hospital, Sukkur, PAK; 4 Internal Medicine, Jinnah Sindh Medical University, Karachi, PAK

**Keywords:** hepatitis b, rict, serology

## Abstract

Introduction: In low-income and high hepatitis B and C virus burden countries like Pakistan, it is important to develop cheap yet efficient strategies in diagnosing as well as treating hepatitis. The aim of this study is to assess the sensitivity and specificity of serum hepatitis B surface antigen (HbsAg) via Rapid Immunoassay Chromatographic Test (RICT) for the screening of hepatitis B, compared to the gold standard laboratory-based method.

Methods: The study was conducted in the Hepatology Clinic of Civil Hospital, Sukkur. All records of the clinic from June 1, 2018, to December 31, 2018, were accessed for identification of the records in which hepatitis B screening via RICT and then confirmatory polymerase chair reaction (PCR) by gene amplification with forward and reverse primers was done.

Results: There were 151 samples in this study. There were 32 (94.1%) true-positive and three (5.8%) false-negative samples. There were two (2.5%) false-positive and 114 (97.4%) true-negative samples. The sensitivity of HbsAg detection via RICT for the screening of 1-1B V was 91.43%, specificity was 98.28% and the accuracy was 96.69%, compared to PCR.

Conclusion: The RICT method has high sensitivity and specificity. In low-income and high-hepatitis B virus-burden countries like Pakistan, it serves as a very efficient screening tool that is easy to use, cheaper in cost, and gives rapid and accurate results.

## Introduction

Globally, around two billion people are infected with hepatitis B virus (HBV) [1]. Out of these, four hundred million are suffering from chronic HBV infection [2]. In Pakistan, as many as nine million people are infected with HBV [3,4]. This endemic situation in Pakistan has been explained by lack of proper healthcare facilities, poverty, and lack of awareness regarding its transmission [2].

Serological markers for hepatitis B include hepatitis B surface antigen (HbsAg), hepatitis B surface antibody (anti-HBs), hepatitis B e-antigen (HBeAg), hepatitis B e-antibody (anti-HBe), and hepatitis B core antibodies (anti-HBc IgM and IgG). These serological markers are used to identify patients with HBV infection, to predict the natural course of chronic hepatitis B (CHB), to note the clinical stages of hepatitis B, and to monitor the efficacy of treatment given [5].

Among these serological markers, HbsAg is considered as the hallmark of HBV infection. It takes one to 10 weeks for HbsAg to appear positive in blood after acute exposure to HBV, even before serum liver function tests becomes abnormal or the patient exhibits clinical symptoms of hepatitis. If HbsAg persists for more than six months, it indicates chronic infection [6]. After the resolution of hepatitis B, HbsAg becomes negative shortly after illness subsides. Hence, HbsAg plays a crucial role in screening healthy individuals for HBV with no clinical, laboratory, biochemical, or histological findings of chronic liver disease [7]. HbsAg can be detected using various techniques. One of them is rapid diagnostic tests (RDTs) in lateral flow, flow-through or simple agglutination assays formats. Laboratory-based immunoassays to detect HBsAg include the conventional radioimmunoassay, enzyme immunoassays, and also newer technology such as electrochemiluminescence. [8]. In this study, the accuracy, sensitivity, and specificity of HBV screening by detecting HbsAg via Rapid Immunoassay Chromatographic Test (RICT) are evaluated as compared to the confirmatory polymerase chain reaction (PCR).

## Materials and methods

The study was conducted in the Hepatology Clinic of Civil Hospital, Sukkur. All clinic records from June 1, 2018, to December 31, 2018, were accessed for identification of records in which hepatitis B screening via RICT (Determine; Abbott, Chicago, IL, USA) and then confirmatory PCR by gene amplification in forward and reverse primers (Macrogen, Seoul, Korea) was done. There were 151 such samples and all of these were included in the study. Those who were positive for HbsAg on screening were termed “screening positive” and the samples which were negative for HbsAg on screening were termed as “screening negative.” PCR was done for all samples. Samples that were positive on screening and then also positive on PCR were termed as “true positive,” those which were positive on screening but negative on PCR were termed as “false positive,” samples which were negative on screening but were positive on PCR were termed as “false negative,” and samples which were negative on both screening and PCR were termed as “true negative.” Data were entered in Microsoft Excel and frequencies and percentages were calculated. A diagnostic test evaluation calculator on MedCalc (https://www.medcalc.org/) was used to determine sensitivity, specificity, accuracy, and positive and negative predictive value [9].

## Results

There were 151 samples in this study. There were 34 (22.5%) samples that were positive for HBV on screening and 117 (77.5%) were negative. When a confirmatory test was done, there were 32 (94.1%) true positive and three (5.8%) false-negative samples. There were two (2.5%) false-positive and 114 (97.4%) true negative samples as shown in Table 1.

**Table 1:** Screening results N = number

Screening Positive (N=34)		Screening Negative (N=117)	
True-Positive	False-Positive	False-negative	True-negative
32 (94.1%)	2 (5.8%)	3 (2.5%)	114 (97.4%)

The sensitivity of HbsAg detection via RICT for the screening of HBV was 91.43% in this study. The specificity was 98.28% and accuracy was 96.69% (Table 2).

**Table 2:** Sensitivity and Specificity Analysis CI = Confidence Interval

	Value	95% CI
Sensitivity	91.43%	76.94% to 98.20%
Specificity	98.28%	93.91% to 99.79%
Positive Predictive Value	94.12%	80.14% to 98.45%
Negative Predictive Value	97.44%	92.79% to 99.12%
Accuracy	96.69%	92.44% to 98.92%

## Discussion

For the longest time, HbsAg (hepatitis B surface antigen) has been used as the screening modality of hepatitis B virus and is considered of pivotal importance by World Health Organization (WHO) [10]. In countries with a low prevalence of HBV, detection of anti-HBc (hepatitis B core antibody) has added to HbsAg as a screening modality. In countries with medium/high prevalence of HBV infection, besides HbsAg, tests for HBV deoxyribonucleic acid (DNA) have been implemented, usually together with equivalent tests for HCV ribonucleic acid (RNA) and HIV RNA [11].

In this study, HbsAg has shown high sensitivity, specificity, and accuracy for the hepatitis B virus. A meta-analysis of 33 studies [12] has shown pooled sensitivity and specificity of 90.0% (95% CI: 89.1-90.8) and 99.5% (95% C1:99.4-99.5), respectively, which is comparable to the outcomes of this study. Comparatively, in a metanalysis from Korea, which is a high burden country in terms of HBV, the pooled sensitivity and specificity of serum HbsAg on RICT was 98.07% (95% CI:97.67-98.47%) and 99.56% (95%CI:99.21-99.91%), respectively [13].

Research has also associated positive serum HbsAg levels with transcription activity of covalently closed circular DNA (cccDNA) [14-16]. The difference in the serum HbsAg levels during the different phases of infection indicates the distribution of cccDNA during the respective phases of the disease. Serum HBsAg titers are higher in patients with HBeAg-positive chronic hepatitis B than those with HBeAg-negative chronic hepatitis [15-17]. Monitoring of quantitative HBsAg levels predicts treatment response to interferon and disease progression in HBeAg-negative chronic hepatitis B patients with normal serum alanine amine aminotransferase levels [18,19].

RICT is easier to handle and cheaper in cost which is why almost all healthcare facilities use it in lieu of the gold standard enzyme-linked immunosorbent assay (ELISA). Although this study did not compare its RICT results with ELISA, which is one of its limitations, other studies from Pakistan have shown comparable results of both methods [20].

## Conclusions

Overall, the RICT method has high sensitivity and specificity. In low-income and high-HBV-burden countries like Pakistan, it serves as a very efficient screening tool that is easy to use, cheaper in cost, and gives rapid and accurate results. It is recommended to conduct large scale studies to compare various brands of RICT and ELISA kits available in Pakistan to gauge their efficiency. Clinicians should eagerly use the RICT method for the detection of hepatitis B and C in their patients, as it provides a cheap and accurate alternative diagnosis for hepatitis B.
